# The Book World of Medicine and Science

**Published:** 1893-07-15

**Authors:** 


### The Book World of Medicine and Science.

The Health Resorts of Europe. A Medical Guide to the Mineral Springs, Climatic, Mountain, and Seaside Health Resorts, Milk, Whey, Grape, Earth, Mud, Sand, and Air Cures of Europe. By Thomas Linn, M.D., with a preface by E. A. Sanson, M.D. (Henry Kimpton. 1893.)

Dr. Linn states that the treatment of acute disease has much improved, but that "neither the *materia medica*, nor the newer surgical operations have much value in the relief or cure of those constitutional states called chronic maladies." For these Dr. Linn thinks the best forms of treatment are found in mineral waters, climatic resorts, sea baths, and other hygienic therapeutical agents. In the introductory observations, the choice of a watering-place is considered, and the first counsel given is to see a physician before determining, and not to take the advice of some friend who may have been benefited by going to a certain place. "The haphazard, unscientific selection of mineral-spring waters is the cause of a vast amount of complication in diseases of a chronic nature." The author also mentions the desirability of preliminary treatment. "In the old times no one went to a thermal or mineral water station without first going through a medicinal course of preparatory treatment. People nowadays are quite indifferent to this; but the omission is an error." The so-called exercise cure, grape cure, milk and whey cure, and sand or earth cure, are so briefly noticed that their mention might well have been avoided in the secondary title of the book. *Paras* are inserted, showing what useful articles should be carried about, among which are enumerated a spirit lamp, cognac, and a pocket compass, for the landlord will often declare his rooms face in a different direction to what they really do. Dr. Linn has no faith in bottled mineral waters, because a "mineral water, be it ever so well corked and bottled, will be found perfectly good only for the first glass." This statement may be questioned, especially with reference to some of the bottled aperient mineral waters, Hunyadi Janos for instance. Nevertheless, experience has fully demonstrated, that to obtain the full benefit from mineral waters they ought to be taken in conjunction with change of scene, society, occupation, or amusement, and of diet. Next we have a classification of mineral waters, which Dr. Linn divides into sulphuretted, saline, alkaline, and ferruginous. It is also briefly mentioned what classes of invalids should use these different waters. A description of health resorts by countries runs through the greater part of the book. Of these we will only refer to some in Great Britain. So far as medicinal value is concerned there are in this country (although Dr. Linn does not say so) mineral waters equal to any found abroad, although so much cannot be advanced in favour of the climate. Harrogate, for instance, affords sulphuretted, saline, and chalybeate waters, which for anæmia, chlorosis, gout, rheumatism, &c., are as good as can be taken. Droitwich has brine waters, not surpassed for rheumatism, gout, scrofulous, and some skin affections. Then there is Bath and Tunbridge Wells. It may, however, be admitted that on the whole Continental health resorts are better fitted and rendered more agreeable to sojourners than is the case in this country. At the end of Dr. Linn's book there is a list of various London

**THE BOOK WORLD OF MEDICINE AND SCIENCE.**—(*continued*).

physicians and specialists which should not have found a place. It is also questionable whether Dr. Sanson's preface should have appeared. For the book requires no such commendation as that given by Dr. Sanson, being fully adapted to stand on its own merits. It may be accepted as what it professes to be, viz., a guide to the health resorts of Europe. Whether, having in recollection the number of books published on the same subject, another were required, is a different question.

### THE JULY REVIEWS.

In the NINETEENTH CENTURY Mrs. King has a pleasant little article on a mediæval work translated from the German in 1561, "A most excellente and perfecte homish apothecarye or physicke book, for all the grefes and diseases of the body." Some of the methods of dealing with madness may be new to our readers. "He that is become mad with sadness ought to be fayre spoken, and many things should be promised him and *some be given*. If it commeth of Flegma, then are hys braines corrupt, and to such an one doth the devill glady accompany: hys best meates were old hennes or cockes well sodden. If a manne's wittes were spred abroad, and thou wilt gather agayn the scattered wits, then take a greate brasse basin and set it sidelings to the wall, so that it doe lean wholly upon the wall, and take a laver wyth a cock, full of water; set that high upon a cupborde, and open the cock a litle, so that the water drop by litle and litle upon the basin, and make a ringinge, and run out of the basin agayne. Into this chamber lay the patient so that he cannot see this; then doth he muse so much upon that droppinge and ringinge, what it may be, that at the last he fastneth his wittes and gathereth them agayn."

Dr. Jessopp's protest against the abuse of the expression "Robbing God" as applied indiscriminately to Church disendowment should be read by everyone interested in this burning question. Holding strong views on the subject himself, his attitude towards it might, nevertheless, serve as a model no less for one party than the other.

